# Prenatal and perinatal risks for late language emergence in a population-level sample of twins at age 2

**DOI:** 10.1186/s12887-018-1035-9

**Published:** 2018-02-07

**Authors:** Catherine L. Taylor, Mabel L. Rice, Daniel Christensen, Eve Blair, Stephen R. Zubrick

**Affiliations:** 1 Telethon Kids Institute, 100 Roberts Rd, Subiaco, WA 6008 Australia; 2 The University of Western Australia, 35 Stirling Highway, Nedlands, WA 6009 Australia; 3 University of Kansas, Dole Human Development Center, 1000 Sunnyside Avenue, Lawrence, KS 66045-7555 USA

**Keywords:** (5, Max 10): Twins, Language, Late language emergence, Child development, Australia

## Abstract

**Background:**

Late Language Emergence (LLE) in the first two years of life is one of the most common parental concerns about child development and reasons for seeking advice from health professionals. LLE is much more prevalent in twins (38%) than singletons (20%). In studies of language development in twins without overt disability, adverse prenatal and perinatal environments have been reported to play a lesser role in the etiology of LLE than adverse postnatal environments. However, there is a lack of population-level evidence about prenatal and perinatal risk factors for LLE in twins. This study investigated the extent to which prenatal and perinatal risk factors were associated with LLE in a population-level sample of twins at age 2 without overt disability.

**Methods:**

The sample comprised 473 twin pairs drawn from a population sample frame comprising statutory notifications of all births in Western Australia (WA), 2000–2003. Twin pairs in which either twin had a known developmental disorder or exposure to language(s) other than English were excluded. Of the 946 twins, 47.9% were male. There were 313 dizygotic and 160 monozygotic twin pairs. LLE was defined as a score at or below the gender-specific 10th percentile on the MacArthur Communicative Development Inventories: Words and Sentences (CDI-WS) (Words Produced). Bivariate and multivariable logistic regression was used to investigate risk factors associated with LLE.

**Results:**

In the multivariable model, risk factors for LLE in order of decreasing magnitude were: Gestational diabetes had an adjusted odds ratio (aOR) of 19.5 (95% confidence interval (CI) 1.2, 313.1); prolonged TSR (aOR: 13.6 [2.0, 91.1]); multiparity (aOR: 7.6 [1.6, 37.5]), monozygosity (aOR: 6.9 [1.7, 27.9]) and fetal growth restriction (aOR: 4.6 [1.7, 12.7]). Sociodemographic risk factors (e.g., low maternal education, socioeconomic area disadvantage) were not associated with increased odds of LLE.

**Conclusions:**

The results suggest that adverse prenatal and perinatal environments are important in the etiology of LLE in twins at age 2. It is important that health professionals discuss twin pregnancy and birth risks for delayed speech and language milestones with parents and provide ongoing developmental monitoring for all twins, not just twins with overt disability.

## Background

In the first two years of life, children achieve important milestones in language development that are highly anticipated by parents. Children with normal language emergence (NLE) typically start to produce single words around their first birthday. By their second birthday, children with NLE start to combine 2–3 words in simple sentences, signalling the emergence of grammar [1]. The term ‘Late Language Emergence’ (LLE) is used to describe toddlers who, despite otherwise healthy development, do not meet age expectations for receptive and/or expressive language development at 24 months [2]. Failure to attain these milestones are ‘red flags’ for referral to a developmental paediatrician [3].

LLE is a common condition, with population-level estimates for singletons ranging from 13%, based on receptive and expressive criterion [2], to 19%, based on expressive language criterion [2, 4]. Our recent population-level estimate for twins was 37.8%, much higher than for singletons. The prevalence of LLE was higher still for monozygotic (MZ) twins compared to dizygotic (DZ twins (46.5% vs. 31.0%) [5] and highly heritable, consistent with the UK Twins Early Development Study (TEDS) [6]. Postnatal environmental influences, in the form of poorer quality maternal interactions, have been positively associated with LLE in twins [7–9]. A recent study reported genotype-environment correlations between parental language input and twin language development [10].

Population-level studies of twins at age 2 have reported higher mean expressive vocabulary scores for females compared to males [5, 11]. This is consistent with studies of singletons [1, 2, 12] and is attributed to differential neurobiological maturation favouring girls [13]. Because early language development follows a different developmental course in girls and boys, gender-specific norms are used to identify LLE [6].

Twin pregnancies have higher rates of prenatal, perinatal and neonatal mortality and morbidity than singleton pregnancies [14, 15]. Twins’ early mental and motor development, at 6, 12 and 18 months, has been reported to lag behind singletons and to be associated with low birthweight, not family socioeconomic circumstances [16]. Studies have yielded a mixed picture of the relative importance of prenatal and perinatal environment risk factors in the etiology of LLE. Findings have varied across study designs and methods. Studies that have included twins whose birthweight and/or gestational age was in the low range have reported significant associations between prenatal and perinatal risk factors and lower verbal and nonverbal cognitive abilities [17–19]. Whereas, studies that have selected or adjusted for birthweight and/or gestational age have reported negligible associations between prenatal and perinatal risk factors and LLE [15, 20, 21].

The aim of the present study was to investigate prenatal and perinatal contributions to LLE in a longitudinal population-representative sample of twins without overt disability.

## Methods

### Study design and twin sample

The study design was a prospective cohort study of twins drawn from a total population sample frame comprising statutory notifications of all births in Western Australia (WA) in 2000–2003 [22].

There were 1135 sets of live twins born in this time period; 941 (83%) families were contacted by mail, and 698 (74%) consented to participate in the study, 61% of all twins born in WA in 2000–2003. A comparison with data for all twins born in 2000–2003 showed that the study participants were broadly representative of the total twin population from which they were drawn [5]. Twin pairs with exposure to languages other than English (52 twin pairs) or twin pairs in which at least one twin had hearing impairment, neurological disorders, or developmental disorders (14 twin pairs) were excluded from the twin sample. The exclusionary criteria resulted in 633 twin pairs who were eligible to participate in the prospective longitudinal cohort study. A postal questionnaire was sent to the twins’ parents one month prior to the twins’ second birthday. The response rate to the postal questionnaire was 75%. In this study, questionnaire data were available for 473 eligible twin pairs of approximately 2 years of age (in days, mean age is 755.8, range, 687–899). There were 454 boys (47.9%) and 492 girls (52.1%).

### Measures

#### Outcome variable

An Australian adaptation of the MacArthur Communicative Development Inventories: Words and Sentences (CDI-WS) [6] was administered at age 2 by postal questionnaire. With the permission of the authors, 24 Standard American English vocabulary items were replaced with Standard Australian English vocabulary items (e.g., ‘nappy’ for ‘diaper’; ‘footpath for ‘sidewalk’ [5]. This is consistent with Reilly et al. (2009 [12]. LLE was defined as a gender-specific score at or below the 10th percentile on the CDI-WS (Words Produced). This equated to 119 words or less for girls and 79 words or less for boys [23]. NLE was defined as a gender-specific score above the 10th percentile on the CDI-WS (Words Produced) [6]. This is also the criterion that was used by Reilly et al. (2009) to identify LLE in a population-based sample of Australian children at 24 months. The CDI-WS and its adaptations have robust psychometric properties and are the most well recognized reliable, valid and feasible assessments for toddlers [24, 25].

### Predictor variables

#### Maternal variables

The data source for maternal, pregnancy, labour, delivery and neonatal variables was the Midwives’ Notification System (MNS). These data are collected by statute on all live births, stillbirths, and neonatal deaths in WA [22]. MNS variables included the mother’s age in years, height in centimetres, parity, marital status, ethnic status and residential address. The mother’s residential address at the time of the birth of the twins was linked to the 1996 Population and Housing Census. Three small-area indices (Socioeconomic Indicators for Areas: SEIFA) were available for each twin-pair [26]. Each index summarizes a different aspect of the socio-economic conditions of the Australian population using a combination of variables. The Index of Relative Socio-Economic Disadvantage, which is used here, is derived from variables that reflect or measure relative disadvantage. Variables used to calculate the index of relative socio-economic disadvantage include low income, low educational attainment, high unemployment and people with low skilled occupations. Lower scores are associated with greater disadvantage. Maternal education, country of birth and family income variables were collected by postal questionnaire.

#### Pregnancy variables

Pregnancy variables included binary variables to indicate the presence or absence of the following circumstances: threatened abortion, pre-eclampsia, placenta praevia, abruption, antepartum haemorrhage (APH), gestational diabetes, fertility treatment, threatened pre-term labour, precipitate delivery, and post-partum haemorrhage (PPH). We also coded a general category for ‘other pregnancy complications’ which occurred in proportions too small to model.

#### Infant variables

We included several characteristics relevant to the infant’s status at birth. For each infant we included the infant’s gender, twin birth-order and binary indicators for fetal distress, cephalopelvic disproportion, prolapsed cord, 5-min Apgar score, Time to Spontaneous Respiration (TSR), and intubation status.

In addition to these we also included estimated gestational age and a measure of each infant’s proportion of optimal birthweight (POBW). POBW is a measure of the appropriateness of intrauterine growth and is routinely calculated from the birth records of all children born in Western Australia. Because birthweight is the end result of growth over the period of gestation it is therefore determined both by the length of gestation and the rate of intra-uterine growth. Duration of gestation may be curtailed or prolonged, and this is usually the result of pathological factors, hence abnormal duration of gestation may be considered to reflect pathological factors. However, since delivery must follow the period of intrauterine growth, duration of gestation is not a determinant of growth and hence cannot be a pathological determinant of growth, though it is the primary determinant of birthweight.

The rate of intrauterine growth is determined by many factors both pathological (maternal, fetal or environmental) and non-pathological (genetic endowment, particularly fetal gender, and maternal environment). Thus it is appropriate that fetal growth rate should vary between individuals, since the non-pathological factors determining growth rate varies between individuals. For example, female newborns appropriately weigh less than male newborns of the same gestation; babies of small women weigh less than babies of tall women and a woman’s first birth tends to weigh less than her subsequent births. We define the optimal fetal growth rate for any particular fetus as the median birthweight achieved by fetuses with the same values for the non-pathological determinants of fetal growth and duration of gestation, in the absence of any pathological determinants of fetal growth. This median is expressed as the ‘optimal birthweight’ once the values of the non-pathological determinants of growth have been specified.

The non-pathological determinants considered in our statistical models of POBW were fetal gender, maternal age, height and parity. Exclusion of pathological factors was achieved by limiting the sample from which optimal birthweights were identified to singleton, live births without congenital abnormalities born to non-smoking mothers following pregnancies without any complications known to affect intrauterine growth [27]. The median value of POBW is 100 and values less than this signify infants that are under grown while values greater than this represent growth in excess of optimal growth.

In this study POBW and gestational age were defined as ‘at risk for twins’. For POBW this was defined as the bottom 15% of the study sample (a POBW of ≤ 76.43), and for gestational age this was defined as gestational age of 33 weeks or less.

#### Zygosity

Twin zygosity was determined by molecular analysis of buccal swab samples. For twin pairs with unknown zygosity, a discriminant analysis of questionnaire items reported by parents was used to assign zygosity. The final twin counts were 313 DZ pairs and 160 MZ pairs, for a total of 473 pairs and 946 individuals [5].

Table 1 indicates that there are a number of candidate predictors with small numbers of children in the ‘at risk’ categories. Although it is important to describe the distribution of these predictors within the twin population, some of these predictors contained so few children they were considered unsuitable for the logistic regression analyses which follow, and were excluded from further consideration. These predictors were: abruption, placenta praevia, precipitate delivery, intubation, cephalopelvic disproportion, prolapsed cord, and 5-min Apgar less than 7.

**Table 1:** Risk factors for LLE in twins at age 2

	LLE (*n* = 358)	NLE (*n* = 588)	Bivariate	Multivariable (*N* = 894)
Characteristic	N (%)	N (%)	OR [95% CI]	aOR [95% CI]
Maternal				
Maternal age				
<=20)	5 (1.4%)	13 (2.2%)	0.1 [0, 12]	0.6 [0.0, 68.8]
21-25	32 (8.9%)	52 (8.8%)	0.7 [0.1, 5.3]	0.7 [0.1, 7.2]
26-30	120 (33.5%)	170 (28.9%)	1.0 [referent]	1.0 [referent]
31-35	133 (37.2%)	219 (37.2%)	0.6 [0.2, 2.4]	0.7 [0.2, 2.8]
36-40	63 (17.6%)	123 (20.9%)	0.4 [0.1, 1.8]	0.4 [0.1, 2.2]
>40	5 (1.4%)	11 (1.9%)	0.3 [0.0, 22.2]	0.4 [0.0, 62.4]
Maternal education				
<12 years	73 (20.4%)	123 (21%)	2.6 [0.5, 12.3]	0.7 [0.1, 4.3]
12 years	97 (27.1%)	107 (18.3%)	9.6 [2.0, 45.7]**	3.4 [0.6, 19.7]
Post school study	23 (6.4%)	55 (9.4%)	0.9 [0.1, 0 8]	2.5 [0.2, 28.5]
Trade certificate	78 (21.8%)	104 (17.7%)	5.2 [1.0, 26.1]*	2.3 [0.4, 13.9]
Completed post school qualification	87 (24.3%)	197 (33.6%)	1.0 [referent]	1.0 [referent]
Mother’s country of birth				
Australia	288 (80.4%)	440 (74.8%)	1.0 [referent]	1.0 [referent]
NZ+UK	33 (9.2%)	69 (11.7%)	0.3 [0.1, 2.2]	0.5 [0.1, 4.0]
Not known	21 (5.9%)	33 (5.6%)	0.9 [0.1, 9.7]	0.6 [0.0, 17.2]
Other	16 (4.5%)	46 (7.8%)	0.1 [0.0, 1.6]	0.1 [0.0, 1.7]
Marital status				
Married	336 (96.6%)	556 (96.5%)	1.0 [referent]	1.0 [referent]
Single, widowed, divorced	12 (3.4%)	20 (3.5%)	1 [0.0, 21.2]	2.6 [0.1, 77.1]
Income				
$200 to $399 per week	10 (2.8%)	22 (3.7%)	0.8 [0.0, 26.8]	0.3 [0.0, 21.1]
$400 to $599 per week	25 (7%)	59 (10%)	0.8 [0.1, 8.8]	0.4 [0.0, 5.7]
$600 $799 per week	50 (14%)	90 (15.3%)	1.8 [0.2, 14.2]	1.2 [0.1, 12.6]
$800 to $999 per week	58 (16.2%)	62 (10.5%)	8.9 [1.0, 76.8]*	6.5 [0.6, 73.0]
$1,000 to $1,499 per week	87 (24.3%)	161 (27.4%)	1.6 [0.2, 10.1]	1.7 [0.2, 13.7]
$1,500 to $1,999 per week	62 (17.3%)	82 (13.9%)	4.4 [0.6, 35.3]	4.9 [0.5, 47.8]
$2,000 to $2,400 or more per week	40 (11.2%)	86 (14.6%)	1.0 [referent]	1.0 [referent]
Not stated	26 (7.3%)	26 (4.4%)	11 [0.7, 172.7]	13.1 [0.6, 277.2]
Socio-economic area disadvantage^a^				
<15th percentile of sample	62 (17.3%)	82 (13.9%)	2.2 [0.5, 10.4]	1.7 [0.3, 8.9]
>15^th^ percentile of the sample	296 (82.7%)	506 (86.1%)	1.0 [referent]	1.0 [referent]
Parity				
0	91 (26.1%)	229 (39.8%)	1.0 [referent]	1.0 [referent]
1	133 (38.2%)	179 (31.1%)	7.4 [1.8, 29.4]**	7.6 [1.6, 37.5]**
>2	124 (35.6%)	168 (29.2%)	7.2 [1.8, 29.3]**	7.9 [1.5, 41.9]**
Height				
lowest tercile	122 (34.1%)	176 (29.9%)	1.5 [0.4, 5.9]	0.8 [0.2, 3.6]
middle tercile	122 (34.1%)	226 (38.4%)	0.7 [0.2, 2.6]	0.6 [0.1, 2.6]
highest tercile	114 (31.8%)	186 (31.6%)	1.0 [referent]	1.0 [referent]
Pregnancy				
Threatened abortion				
No	329 (91.9%)	547 (93%)	1.0 [referent]	1.0 [referent]
Yes	29 (8.1%)	41 (7%)	1.7 [0.2, 13.7]	1.3 [0.1, 14.1]
Pre-eclampsia				
No	306 (85.5%)	490 (83.3%)	1.0 [referent]	1.0 [referent]
Yes	52 (14.5%)	98 (16.7%)	0.6 [0.1, 2.8]	0.6 [0.1, 3.0]
Placenta praevia^b^				
No	354 (98.9%)	588 (100%)		
Yes	4 (1.1%)	0 (0%)		
Abruption^b^				
No	355 (99.2%)	587 (99.8%)		
Yes	3 (0.8%)	1 (0.2%)		
APH				
No	346 (96.6%)	568 (96.6%)	1.0 [referent]	1.0 [referent]
Yes	12 (3.4%)	20 (3.4%)	1 [0.0, 20.4]	1 [0.0, 26.4]
Other pregnancy complications				
None	321 (89.7%)	547 (93%)	1.0 [referent]	1.0 [referent]
Other pregnancy complications	37 (10.3%)	41 (7%)	4 [0.5, 28.9]	5.4 [0.6, 45.3]
Gestational Diabetes				
None	333 (93%)	567 (96.4%)	1.0 [referent]	1.0 [referent]
Diabetes	25 (7%)	21 (3.6%)	9.6 [0.8, 122.2]	19.5 [1.2, 313.1]*
Fertility treatments				
No	287 (82.5%)	421 (73.3%)	1.0 [referent]	1.0 [referent]
Yes	61 (17.5%)	153 (26.7%)	0.2 [0.0, 0.8]*	0.5 [0.1, 2.4]
Threatened preterm labour				
None	322 (89.9%)	546 (92.9%)	1.0 [referent]	1.0 [referent]
Threatened preterm labour	36 (10.1%)	42 (7.1%)	3.3 [0.4, 24.2]	2.5 [0.3, 21.4]
Precipitate delivery^b^				
None	349 (97.5%)	585 (99.5%)		
Yes	9 (2.5%)	3 (0.5%)		
Fetal distress				
None	317 (88.5%)	531 (90.3%)	1.0 [referent]	1.0 [referent]
Fetal distress	41 (11.5%)	57 (9.7%)	1.2 [0.2, 6.3]	0.6 [0.1 , 3.6]
Cephalopelvic disproportion^b^				
None	356 (99.4%)	586 (99.7%)		
Cephalopelvic disproportion	2 (0.6%)	2 (0.3%)		
Prolapsed cord^b^				
None	356 (99.4%)	581 (98.8%)		
Prolapsed cord	2 (0.6%)	7 (1.2%)		
PPH >500mls				
No	287 (80.2%)	483 (82.1%)	1.0 [referent]	1.0 [referent]
Yes	71 (19.8%)	105 (17.9%)	1.5 [0.4, 6.2]	1.7 [0.4, 8.4]
Infant				
Gender^c^				
Male	173 (48.3%)	281 (47.8%)		
Female	185 (51.7%)	307 (52.2%)		
Zygosity				
DZ	204 (57%)	422 (71.8%)	1.0 [referent]	1.0 [referent]
MZ	154 (43%)	166 (28.2%)	7.4 [2.3, 24]***	6.9 [1.7, 27.9]**
Birth order				
First-born twin	179 (50%)	294 (50%)	1.0 [referent]	1.0 [referent]
Second-born twin	179 (50%)	294 (50%)	1 [0.6, 1.6]	0.9 [0.5, 1.5]
Apgar 5-minutes <7^b^				
No	341 (98%)	574 (99.7%)		
Yes	7 (2%)	2 (0.3%)		
Total	348 (100%)	576 (100%)		
TSR > 2 minutes				
No	311 (92.3%)	542 (96.6%)	1.0 [referent]	1.0 [referent]
Yes	26 (7.7%)	19 (3.4%)	13.4 [2.3, 77.7]**	13.6 [2.0, 91.1]**
Intubation^b^				
No	337 (96.8%)	561 (97.4%)		
Yes	11 (3.2%)	15 (2.6%)		
Estimated gestational age				
>34 weeks)	281 (80.7%)	489 (84.9%)	1.0 [referent]	1.0 [referent]
<34weeks	67 (19.3%)	87 (15.1%)	2.6 [0.6, 11.5]	3.2 [0.6, 17.3]
POBW				
normal	275 (79%)	510 (88.5%)	1.0 [referent]	1.0 [referent]
<15th percentile of sample	73 (21%)	66 (11.5%)	6.6 [2.4, 18.1]***	4.6 [1.7, 12.7]**

^a^= defined as bottom 15% of study sample; ^b^= excluded from logistic estimate due to small n.; ^c^ = excluded from logistic estimate as gender is taken into account when defining language delay.

^*P , .05. **P , .01. ***P , .001.^

### Statistical analyses

Our outcome measure (i.e., LLE) was a score at or below the gender-specific 10th percentile for Word Produced on the CDI-WS. Because the outcome measure was gender-specific, gender was not included in the models estimated below.

All predictor variables were modelled as risk variables (e.g., POBW <15th percentile of the sample). For each risk variable, the ‘least risk’ category (e.g., normal POBW) was the reference category (see Table 1). To estimate the odds of LLE, a generalised linear mixed model with a logistic link function was used to explicitly account for the paired structure of the data, and estimate the subject-specific risks for LLE. To account for correlation within twin-pairs, twin-pair specific parameters were estimated by incorporating a random effects component for the twin-pair [28]. These analyses were undertaken in PROC GLIMMIX in SAS version 9.4 [29], using maximum likelihood with adaptive quadrature estimation. For the purposes of simplicity, this analysis is referred to as a logistic regression analysis, as we are estimating the odds of LLE for the candidate predictors. This analysis produced subject-specific odds ratios for LLE. Unadjusted odds ratios (ORs), adjusted odds ratios (aORs), and 95% confidence intervals (CIs) were estimated with bivariate and multivariable logistic regression to identify factors associated with LLE in the study sample.

## Results

Table 1 shows the adjusted and unadjusted odds of LLE associated with the predictor variables. Of 21 maternal, pregnancy, delivery and neonatal risk factors considered, 5 had statistically significant associations with LLE in the multivariable model. In order of odds ratio, from highest to lowest the risk factors were: Gestational diabetes (aOR: 19.5 [1.2, 313.1]), TSR greater than 2 min (aOR: 13.6 [2.0, 91.1]), parity of 1 (aOR: 7.6 [1.6, 37.5]; parity of 2 or more (aOR:7.9 [1.5, 41.9]), monozygosity (aOR: 6.9 [1.7, 27.9]) and POBW below the 15th percentile of the sample (aOR: 4.6 [1.7, 12.7]). The model included maternal sociodemographic risk factors (e.g., low maternal education, socioeconomic area disadvantage) that were not associated with increased odds of LLE.

## Discussion

Late language emergence has long been regarded as the hallmark individual difference between twins and singletons. Large-scale population-level studies have drawn attention to the neurobiological etiology of LLE in singletons [2, 12] and twins at age 2 [5, 11]. Recent population-level behavior genetics studies have drawn attention to the important role of genetic factors in the etiology of LLE in twins [5, 11]. This study has drawn attention to five risk factors for LLE that can be detected and treated by clinicians in the prenatal, perinatal and neonatal periods in twins without frank disability. The benefits of early intervention should translate to reduced risk for LLE at age 2. The current study selected on twins without frank disability but did not select on or control for birthweight and/or gestational age variation. This meant that the independent risk conferred by birthweight, gestational age and fetal growth restriction was quantified in a model that included pregnancy and birth risks as well as sociodemographic risks. Necessarily, studies of twin-singleton differences [19, 20, 30] have selected on or controlled for birthweight and/or gestational age variation between twins and singletons to elucidate mediators and moderators of twinning effects on LLE [21].

The results of this study have drawn attention to the role of gestational diabetes, prolonged TSR, fetal growth restriction in the etiology of LLE. These risks are all well-known complications of twin pregnancy [15, 31] and risk factors for LLE in singletons. This study has shown the pervasive adverse influence of these risks on twins’ neurodevelopment in the second year of life. Prenatal life is a critical phase of brain development, during which even subtle differences in fetal growth have been associated with differences in postnatal brain maturation and cognitive abilities in twins [32].

Multivariate analysis yielded the following significant predictors of LLE in twins, in order of odds ratio from highest to lowest: Gestational diabetes; TSR > 2 min; multiparity; monozygosity and POBW below the 15th percentile of the twin sample. The only risk factor unique to twin pregnancies was monozygosity. This risk factor retained statistical significance in a model that multivariately adjusted for the effects of other risk factors. This suggests that the biological mechanisms underlying MZ twinning itself may contribute to the elevated prevalence of LLE in MZ twins, compared to DZ twins [5], that cannot be attributed to a shared postnatal environment, which all twins share, irrespective of zygosity [7, 10, 33]. The only family environment risk factor was multiparity (i.e., ≥ 1 biological sibling). It was striking to see that the presence of one or more siblings was a risk exposure for LLE in twins, entirely consistent with birth order effects for LLE in singletons [2, 12, 34].

POBW is a population-based estimate of fetal growth that is a more differentiated measure of fetal growth than absolute birthweight. POBW is an important index of the child’s developmental status [2, 35]. The advantage of this measure of appropriateness of growth, over birthweight, is that it is individualised and takes into account the duration of gestation. The advantage over the commonly used percentile measures (sometimes termed ‘small for gestational age’) is that it is more accurate and generalizable at the extremes, and being a parametric ratio quantity, is more amenable to statistical manipulation. The results of this study support the view that where POBW can be calculated, it is generally preferable to more traditional measures such as gestational age and birthweight [36].

### Strengths and limitations

Strengths of the study include the population-based prospective cohort design; use of a reference-group based definition of LLE; use of maternal, pregnancy, labour, delivery and neonatal variables collected prospectively by statute; use of a population-based estimate of fetal growth; and exclusion of twins with developmental disorders. The main limitation of the study was the relatively low prevalence of some of the risk factors, leading to wide CIs for some of the estimates. Another limitation is that the MNS does not include data on pregnancy complications that are unique to twin pregnancies (e.g., twin reversed arterial perfusion and twin-twin transfusion syndrome).

Follow-up investigations are needed to find out if complications in the fetal and neonatal periods play a role in the course of twins’ language development over time.

## Conclusions

The results provided evidence for the role of complications in the fetal and neonatal periods, and monozygotic twinning in the etiology of LLE in twins with otherwise healthy development at age 2. The results draw attention to the importance of optimising prenatal life for twins to counter adverse neurodevelopmental outcomes in the postnatal period.

## Abbreviations

aOR: Adjusted Odds Ratio
APH: Antepartum Haemorrhage
CDI-WS: MacArthur Communicative Development Inventories: Words and Sentences
CIs: Confidence Intervals
DZ: Dizygotic
LLE: Late Language Emergence
MNS: Midwives’ Notification System
MZ: Monozygotic
NLE: Normal Language Emergence
OR: Unadjusted Odds Ratio
POBW: Proportion of Optimal Birthweight
PPH: Post-Partum Haemorrhage
SEIFA: Socioeconomic Indicators for Areas
TEDS: Twins Early Development Study
TSR: Time to Spontaneous Respiration
WA: Western Australia
